# Factors Associated With Influential Health-Promoting Messages on Social Media: Content Analysis of Sina Weibo

**DOI:** 10.2196/20558

**Published:** 2020-10-09

**Authors:** Qingmao Rao, Zuyue Zhang, Yalan Lv, Yong Zhao, Li Bai, Xiaorong Hou

**Affiliations:** 1 College of Medical Informatics Chongqing Medical University Chongqing China; 2 Medical Data Science Academy Chongqing Medical University Chongqing China; 3 School of Public Health and Management Chongqing Medical University Chongqing China; 4 Hospital of Zigong Mental Health Central Zigong China

**Keywords:** health-promoting messages, social media, Sina Weibo, influence, framing effects, health communication

## Abstract

**Background:**

Social media is a powerful tool for the dissemination of health messages. However, few studies have focused on the factors that improve the influence of health messages on social media.

**Objective:**

To explore the influence of goal-framing effects, information organizing, and the use of pictures or videos in health-promoting messages, we conducted a case study of Sina Weibo, a popular social media platform in China.

**Methods:**

Literature review and expert discussion were used to determine the health themes of childhood obesity, smoking, and cancer. Web crawler technology was employed to capture data on health-promoting messages. We used the number of retweets, comments, and likes to evaluate the influence of a message. Statistical analysis was then conducted after manual coding. Specifically, binary logistic regression was used for the data analyses.

**Results:**

We crawled 20,799 Sina Weibo messages and selected 389 health-promoting messages for this study. Results indicated that the use of gain-framed messages could improve the influence of messages regarding childhood obesity (*P*<.001), smoking (*P*=.03), and cancer (*P*<.001). Statistical expressions could improve the influence of messages about childhood obesity (*P*=.02), smoking (*P*=.002), and cancer (*P*<.001). However, the use of videos significantly improved the influence of health-promoting messages only for the smoking-related messages (*P*=.009).

**Conclusions:**

The findings suggested that gain-framed messages and statistical expressions can be successful strategies to improve the influence of messages. Moreover, appropriate pictures and videos should be added as much as possible when generating health-promoting messages.

## Introduction

### Overview

Television, newspapers, radio, magazines, and other traditional media have long been the communication tools relied upon for health communication. More recently, social media, such as Facebook, Twitter, and Sina Weibo (or Weibo), has gained explosive growth, especially in China [1]. As of June 2019, China has 854 million internet users, the vast majority of whom obtain information through social media [2]. An increasing number of scholars believe that social media has great potential as a tool in the field of health care [3] and health promotion [4,5].

Sina Weibo is one of the most popular social media platforms in China. In December 2018, this platform had 462 million active accounts, including more than 37,000 media organizations and 170,000 government agency accounts [6]. Yang et al [1] described Weibo as a mixture of features of Twitter and Facebook. Weibo also has some elements of a bulletin board system, blog, and social networking site. Social media has become a unique platform for health promotion due to its potential for viral messaging [7], its ability to challenge authority [8], and its diversity of users [9]. In China, Weibo has been widely used for health communication [10-12].

However, many health-promoting messages released on social media lack influence [13]. Health-promoting messages transmit health information through mass media to prevent diseases and promote health [14]. Van ’t Riet et al [15] asserted that a health-promoting message should include health-related behaviors and the consequences of behaviors. As a result, health-promoting messages may contain terminologies and substantial expository text [16]. However, Chinese residents have low overall health information literacy [17]. Most people think that health messages on the internet are often too complex to understand [18]. The complex content in health-promoting messages hinders people's willingness to interact with them. Moreover, few studies have explored the effect of using specific communication strategies to enhance people’s participation with health messages on social networking platforms [19].

Some strategies can improve the audience's acceptance of and participation with health messages. Myers [20] believed that health message–framing effects can be conducive to the spread of health-promoting messages and encourage people's health behaviors. Meppelink et al [21,22] used pictures and videos in a health-promoting message to change the communication effect. Allen and Preiss [23] found that a statistical type of information organization made information more persuasive. Sundar [24] suggested that audiences are more likely to recognize information provided by professionals than by nonprofessionals. Social media has broken through the limitations of traditional media and made these strategies easier to use. A previous health information survey on child obesity [25] verified that framing effects could significantly change the audience's attitude toward information. Whether these strategies, especially the framing effects, contribute to the impact of health-promoting messages on the Weibo platform is worth studying.

Weibo has become one of the most notable platforms for people in China to seek health-promoting messages [26]. Examining the factors that shape the degree of influence of health-promoting messages on the Weibo platform is crucial. Many studies on health information dissemination have been carried out by questionnaires, but this technique has the problem of subjective bias. Therefore, this work employed a web crawler and manual coding to collect data from the real-world platform of Weibo. We considered the message-framing types as the influencing factors and explored whether the message sources, expression types, and use of pictures or videos would affect the degree of influence of health-promoting messages. The results of this study can guide the communication of related health themes and provide experimental evidence for theoretical research related to the framing effects of health-promoting messages.

### Background

#### Message-Framing Effects

Kahneman and Tversky [27] first proposed framing effects using the “Asian disease problem” example, thereby beginning the research on framing effects in the field of psychology. Message-framing effects for health-promoting messages have become a hot research topic. Prospect theory can explain framing effects. This theory holds that people can be acutely aware of whether a framing message emphasizes potential benefits or risks [27,28]. Health-promoting messages can be divided into gain-framed messages (which highlight the beneficial consequences of healthy behavior) or loss-framed messages (which underscore the detrimental counterpart) [15]. The gain- and loss-framed effects show that when health care messages emphasize the positive or negative results of an action or omission, the persuasiveness of the messages significantly differ. Previous studies have confirmed that a gain-framed message is effective in promoting the use of sunscreen and exercise activities [29]. Conversely, a loss-framed message is persuasive in promoting mammography, chest self-examination [30], and colorectal cancer detection [31]. Given the prior research [15] and the text-based message expression of Weibo [32], we posit that the gain-framed and loss-framed effects on health-promoting message dissemination on Weibo are similar to those of print media. A Weibo message that clearly expresses positive or negative consequences was regarded as framed.

#### Expression Type and Visuals

A statistical expression message refers to a missive that contains quantitative or numerical information [33]. Many studies have compared the persuasiveness of different types of information organization, especially the statistical and narrative evidence types, albeit with inconclusive results [34,35]. Allen and Preiss [23] believed that the use of statistical expressions in a highly technological world is crucial. Their meta-analysis also indicated that statistical expressions of proof are generally persuasive. A Weibo message that contains numerical evidence is considered a statistical expression message. Visuals [36] refer to adding descriptive pictures or videos to a Weibo message. Through literature review and the research completed by our group [25,37], we found that visuals [38,39] and statistics [39] were two important message features that can be combined with framing effects to affect the influence of health messages. It is easy for users to detect pictures, videos, or precise numbers in Weibo messages, and it is also easy for message creators to add this content to Weibo messages.

#### Evaluation Indicator of the Influence of Health-Promoting Messages in Weibo

In our study, influence was defined as the degree to which a Weibo health-promoting message attracts users to participate in the message interaction, which also evaluates the effectiveness of health communication [40]. Shiratuddin et al [41] and Hassan and Shiratuddin [42] found that retweets, comments, and likes can reflect a user group’s participation and attention to the content of Weibo messages. Retweets constitute the crucial mechanism of message diffusion on Weibo. Retweets are related to a variety of social motivations, such as spreading information to new audiences, pleasing specific audiences, publicly supporting someone, quoting others’ views, and symbolizing friendship, loyalty, or respect. Starbird and Palen [43] believe that retweeting is a kind of information recommendation behavior. Comments and likes are the main ways for users to interact on Weibo. Comments on Weibo refer to users’ personal opinions on a topic, according to their preferences and other subjective demands. A “like” button, represented by a thumbs up symbol, is present at the bottom of every post by the social network users. That symbol is clicked to express love and approval for a particular statement. The “like” option is easy to operate, thereby making the expression convenient and fast.

These 3 behaviors’ costs mainly include time costs and credit costs. The time costs and credit costs for retweets, comments, and likes are different. Compared with the other two operations, the time costs and credit costs of the like operation are the lowest. The comment operation requires the highest time costs and some credit costs. The retweet operation requires few time costs and the highest credit costs. To summarize, we propose that when a Weibo message attracts users to participate in the interaction, the cost of commenting is the highest, retweeting is the second highest, and liking is the lowest. This means that the weights of likes (Lw), retweets (Rw), and comments (Cw) are different for the mathematical expression of the influence score. Therefore, the influence score (ls) of a Weibo message can be defined as the linear weighted sum of the number of retweets, comments, and likes. This can be expressed as:


Is = αLw + βRw + γCw **(1)**

α + β + γ = 1 **(2)**

α < β < γ **(3)**

Xiong et al [44] found that the number of retweets and comments had a positive correlation in a big data study on Weibo messages. The quantitative relationship between β and γ was obtained:


β:γ = 0.84 **(4)**

Based on the data set of messages in the Weibo hot topic list, Wu [45] found the ratio of the number of likes to the sum of the number of retweets and comments in the Weibo messages in which users actively participated in the interaction. The quantitative relationship between α and (β + γ) was obtained:


α:(β + γ) = 0.25 **(5)**

The values of α, β, and γ can be obtained simultaneously with formulas 1, 4, and 5:


Is = 0.2Lw + 0.365Rw + 0.435Cw.

## Methods

### Health Message Themes

Because of the search method on Weibo, we first had to determine the keywords that could represent the health theme in order to search Weibo health messages. To identify health themes, we used the PubMed search engine, using “fram* effects” AND “health message*” as keywords, and obtained 229 papers. A panel of experts discussed health themes from these 229 papers. Through discussion, they found that obesity-related, smoking-related, and cancer-related health themes were mentioned more in the literature and that the public paid high attention to them. Combined with the previous research of our group [25], we ultimately chose “childhood obesity,” “smoking,” and “cancer” as keywords.

Using Python, we wrote a crawler program that could automatically obtain the fields in the Weibo platform according to the given keywords. The resulting fields included the message text, the publisher’s name, and the number of retweets, comments, likes, pictures, and videos.

### Time Ranges of Health-Promoting Messages

**Figure 1:**
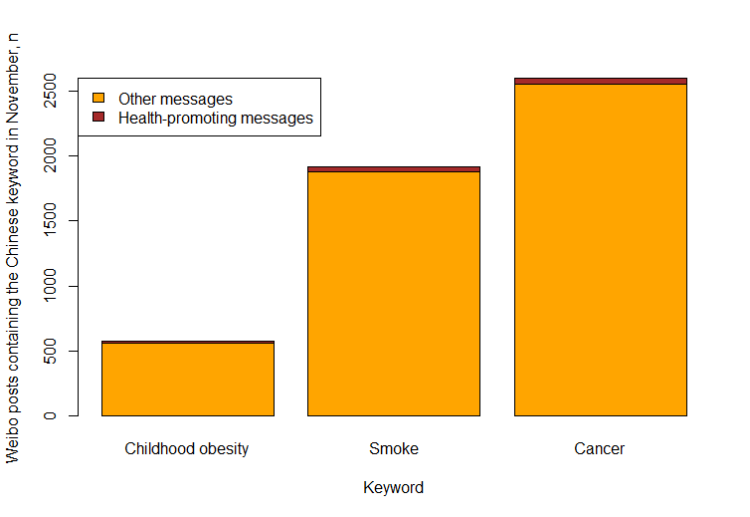
Number of Weibo messages that contained the Chinese keyword in the Weibo database in November 2019. Health-promoting message content had to include health-related behaviors and the consequences of behaviors.

Weibo allows researchers to retrieve messages posted during a specified period through keyword searches. With childhood obesity, smoking, and cancer as the keywords, we retrieved posts for 1 month (November 1 to November 30, 2019). The 2 coders counted the number of health-promoting messages in the search posts and used the Cohen κ coefficient to ensure the consistency between the coders. The statistics of the 2 coders revealed that the proportion of health-promoting messages containing the keywords was 1:4.4:5.1 (childhood obesity:smoking:cancer) (Figure 1). In order to ensure that the number of health-promoting messages in the 3 themes remained similar, we used childhood obesity as the keyword and searched for posts from January 1, 2019, to January 31, 2020 (13 months). Moreover, we employed smoking and cancer as the keywords and searched posts from November 1, 2019, to January 31, 2020 (3 months).

### Coding of Health-Promoting Messages

We used Python's Selenium library to simulate users' log-ins to Weibo's webpage by employing a written crawler code and using Weibo's advanced collection mechanism to search for messages with the keywords. The crawler was designed to output the search results, including the messages; the account names of the messages; the number of retweets, comments, and likes on messages; and any pictures or videos.

**Figure 2:**
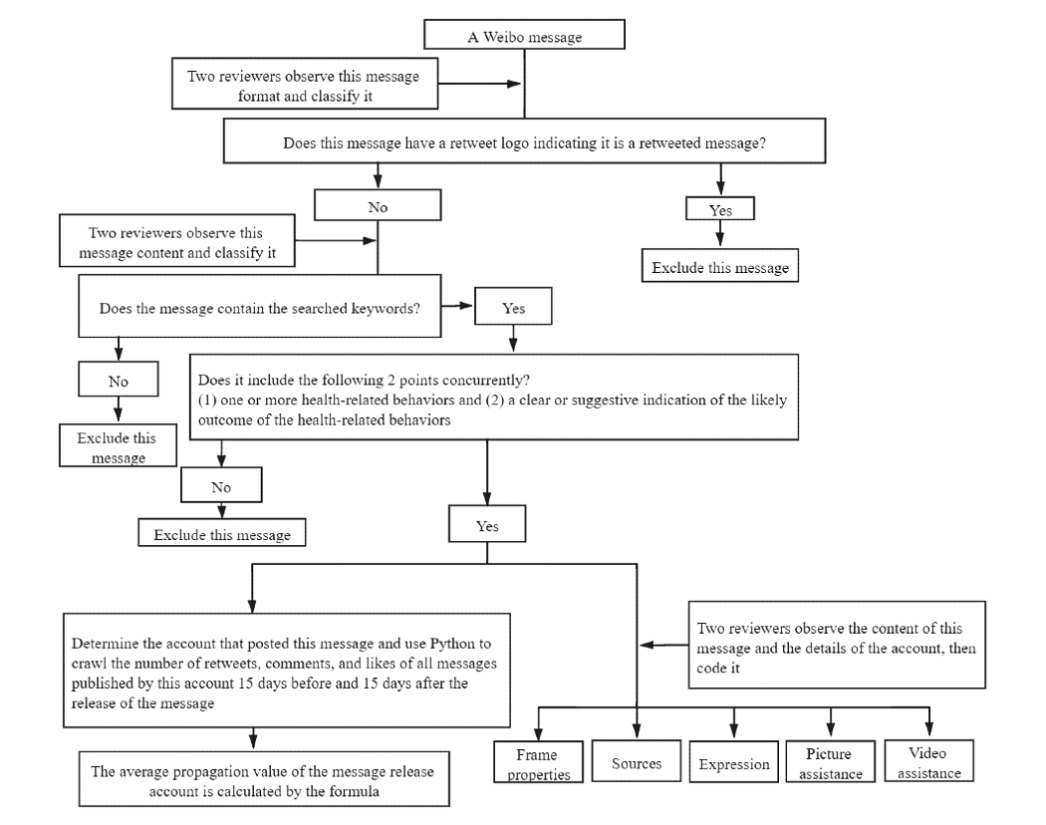
The coding process for the health-promoting messages.

Two reviewers then screened the health-promoting messages from the search results according to the coding process (Figure 2) and coded the degree of influence, frame properties, influence source type, expression type, and presence of pictures and videos, as described in Multimedia Appendix 1 [9,31,45]. The 2 coders studied the coding principles carefully, and pre-experiment coding was carried out. In the pre-experiment coding, the 2 coders communicated effectively. Results that were similarly coded by the 2 reviewers could be entered directly. Messages with different codes were re-examined by a reviewer. Then, after eliminating human error, the coding was submitted to an expert group for judgment if the outcome was inconsistent.

### Statistical Analysis

Data were processed with Excel (Microsoft Corp) software before entry into the database. Data analyses were performed using SPSS 20.0 software (IBM Corp). Frequency and percentage were used to describe the categorical variables of the message characteristics. Binary logistic regression analysis was implemented to evaluate the influencing factors related to the health-promoting messages’ influence. *P* values below .05 were considered statistically significant.

### Quality Control

Before the formal experiment, 2 coders randomly encoded 1800 Weibo messages. Cohen κ was used to measure the consistency in SPSS 20.0. The Cohen κ coefficient of the coders was 0.827 when judging whether a message was a health-promoting message, 0.962 when judging the influence of a health-promoting message, 0.859 when judging the frame properties of a health-promoting message, 0.943 when judging the expression properties of a health-promoting message, 0.977 when judging the sources of a health-promoting message, and 1.000 when judging whether a health-promoting message contained a picture or video. The two coders had good consistency and met the coding requirements of content analysis.

## Results

### Descriptive Statistics of the Characteristics of Health-Promoting Messages

A total of 389 health-promoting messages were included in this study (Table 1). Among these messages, 242 (62.1%) were lower than the average influence score of the disseminator. The chosen items used loss-framed (241/389, 61.8%), gain-framed (127/389, 32.6%), and neutral-framed (21/389, 5.4%) messages. The disseminators of these health-promoting messages were mostly individual users, 31.3% (122/389) of whom possessed professional accounts certified by the platform or were labelled as engaged in the health field and 26.7% (104/389) of whom were not certified by the platform nor labelled as engaged in the health field. Disseminators not from health institutions and organizations (86/389, 22.1%) outnumbered those from health institutions and organizations (77/389, 19.7%). A total of 44.1% (172/389) of the health-promoting messages contained statistical expressions. Furthermore, 51.3% (200/389) of the health-promoting messages added corresponding pictures in addition to the text description. However, only 20.0% (78/389) of the messages added corresponding videos.

**Table 1:** Descriptive statistics of characteristics of health-promoting messages.

Variables		Childhood obesity, n (%) (n=128)	Smoking, n (%) (n=114)	Cancer, n (%) (n=147)	Total, n (%) (N=389)
**Influence degree**					
	High influence	45 (35.2)	36 (28.1)	66 (44.9)	147 (37.7)
	Low influence	83 (64.8)	78 (60.9)	81 (55.1)	242 (62.1)
**Frame properties**					
	Loss framed	74 (57.8)	94 (73.4)	73 (49.7)	241 (61.8)
	Gain framed	48 (37.5)	17 (13.3)	62 (42.2)	127 (32.6)
	Neutral framed	6 (4.7)	3 (2.3)	12 (8.2)	21 (5.4)
**Sources**					
	Ordinary users (health field)	41 (32.0)	28 (21.9)	53 (36.1)	122 (31.3)
	Ordinary users (nonhealth field)	35 (27.3)	29 (22.7)	40 (27.2)	104 (26.7)
	Organizations (health field)	27 (21.1)	29 (22.7)	21 (14.3)	77 (19.7)
	Organizations (nonhealth field)	25 (19.5)	28 (21.9)	33 (22.4)	86 (22.1)
**Expression properties**					
	Statistical expression	54 (42.2)	67 (52.3)	51 (34.7)	172 (44.1)
	Nonstatistical expression	74 (57.8)	47 (36.7)	96 (65.3)	217 (55.6)
**Picture assistance**					
	Yes	77 (60.2)	48 (37.5)	75 (51.0)	200 (51.3)
	No	51 (39.8)	66 (51.6)	72 (49.0)	189 (48.5)
**Video assistance**					
	Yes	15 (11.7)	24 (29.7)	39 (26.5)	78 (20.0)
	No	113 (88.3)	90 (70.3)	108 (73.5)	311 (79.7)

### Binary Logistic Regression of the Information Characteristics and Degree of Dissemination

The influence of each health-promoting message was a dichotomous variable. In this study, we used binary logistic regression to evaluate the impact of framing effects, information sources, expression types, and pictures and videos on the degree of influence of health-promoting messages. The dependent variable of the binary logistic regression model was based on low influence.

We analyzed the 3 focal health message themes and found that the effect of the message characteristics on the message influence did not change with the alteration of the health message themes. For the messages on childhood obesity and cancer, the frame properties and whether the message was a statistical expression had an impact on the message influence. Compared with loss-framed messages, gain-framed messages had a higher degree of message influence (*P*<.001 in childhood obesity and cancer) and used statistical expressions with a higher degree of message influence (*P*=.02 in childhood obesity, *P*<.001 in cancer). In the messages about smoking, the frame properties (whether statistical expressions or otherwise) and the inclusion or exclusion of videos had an impact on the message influence. Compared with the loss-framed messages, the gain-framed messages had a higher degree of message influence (*P*=.03). Messages with statistical expressions had a higher degree of message influence than those with nonstatistical expressions (*P*=.002). Finally, messages with videos had a higher degree of message influence than those without videos (*P*=.009). The effect of the message characteristics on the influence of messages about childhood obesity, smoking, and cancer can be seen in Tables 2-4.

**Table 2:** Binary logistic regression for health-promoting messages regarding childhood obesity.

Parameter		β		SE		Wald chi-square (*df*)		OR^a^ (95% CI)		*P* value	
**Frame properties**											
	Gain framed		–3.210		0.571		31.6 (1)		0.040 (0.013-0.124)		<.001
	Neutral framed		0.280		1.227		0.1 (1)		1.323 (0.119-14.661)		.82
	Loss framed (ref^b^)		N/A^c^		N/A		N/A		N/A		N/A
**Sources**											
	Ordinary users (health field)		–0.072		0.739		0.0 (1)		0.931 (0.219-3.962)		.92
	Ordinary users (nonhealth field)		0.462		0.724		0.4 (1)		1.587 (0.384-6.562)		.52
	Organizations (health field)		–0.958		0.785		1.5 (1)		0.384 (0.082-1.788)		.22
	Organizations (nonhealth field) (ref)		N/A		N/A		N/A		N/A		N/A
**Expression**											
	Statistical expression		–1.227		0.525		5.5 (1)		0.293 (0.105-0.820)		.02
	Nonstatistical expression (ref)		N/A		N/A		N/A		N/A		N/A
**Picture assistance**											
	Yes		–0.162		0.609		0.1 (1)		0.850 (0.258-2.806)		.79
	No (ref)		N/A		N/A		N/A		N/A		N/A
**Video assistance**											
	Yes		–0.334		0.890		0.1 (1)		0.716 (0.125-4.098)		.71
	No (ref)		N/A		N/A		N/A		N/A		N/A

^a^OR: odds ratio.

^b^ref: reference category.

^c^N/A: not applicable.

**Table 3:** Binary logistic regression for health-promoting messages regarding smoking.

Parameter		β	SE	Wald chi-square (*df*)	OR^a^ (95% CI)	*P* value
**Frame properties**						
	Gain framed	–1.412	0.641	4.9 (1)	0.244 (0.069-0.856)	.03
	Neutral framed	–0.163	1.389	0.0 (1)	0.850 (0.850-12.932)	.91
	Loss framed (ref^b^)	N/A^c^	N/A	N/A	N/A	N/A
**Sources**						
	Ordinary users (health field)	0.538	0.687	0.6 (1)	1.713 (0.446-6.580)	.43
	Ordinary users (nonhealth field)	1.209	0.747	2.6 (1)	3.351 (0.776-14.475)	.11
	Organizations (health field)	0.300	0.722	0.2 (1)	1.350 (0.328-5.555)	.68
	Organizations (nonhealth field) (ref)	N/A	N/A	N/A	N/A	N/A
**Expression**						
	Statistical expression	–1.932	0.609	10.1 (1)	0.145 (0.044-0.478)	.002
	Nonstatistical expression (ref)	N/A	N/A	N/A	N/A	N/A
**Picture assistance**						
	Yes	–0.879	0.631	1.9 (1)	0.415 (0.121-1.429)	.16
	No (ref)	N/A	N/A	N/A	N/A	N/A
**Video assistance**						
	Yes	–2.016	0.767	6.9 (1)	0.133 (0.030-0.599)	.009
	No (ref)	N/A	N/A	N/A	N/A	N/A

^a^OR: odds ratio.

^b^ref: reference category.

^c^N/A: not applicable.

**Table 4:** Binary logistic regression for health-promoting messages regarding cancer.

Parameter		β		SE		Wald chi-square (*df*)		OR^a^ (95% CI)		*P* value	
**Frame properties**											
	Gain framed		–2.808		0.482		33.9 (1)		0.060 (0.023-0.155)		<.001
	Neutral framed		–1.019		0.757		1.8 (1)		0.361 (0.082-1.590)		.18
	Loss framed (ref^b^)		N/A^c^		N/A		N/A		N/A		N/A
**Sources**											
	Ordinary users (health field)		0.671		0.587		1.3 (1)		1.955 (0.618-6.183)		.25
	Ordinary users (nonhealth field)		0.461		0.623		0.5 (1)		1.586 (0.468-5.375)		.46
	Organizations (health field)		–0.226		0.745		0.1 (1)		0.798 (0.185-3.436)		.76
	Organizations (nonhealth field) (ref)		N/A		N/A		N/A		N/A		N/A
**Expression**											
	Statistical expression		–1.714		0.473		13.1 (1)		0.180 (0.071-0.455)		<.001
	Nonstatistical expression (ref)		N/A		N/A		N/A		N/A		N/A
**Picture assistance**											
	Yes		–0.286		0.577		0.2 (1)		0.751 (0.242-2.328)		.62
	No (ref)		N/A		N/A		N/A		N/A		N/A
**Video assistance**											
	Yes		–0.109		0.661		0.0 (1)		0.897 (0.245-3.277)		.87
	No (ref)		N/A		N/A		N/A		N/A		N/A

^a^OR: odds ratio.

^b^ref: reference category.

^c^N/A: not applicable.

## Discussion

### Principal Results

To the best of our knowledge, this study has broken new ground in two aspects by (1) exploring the application of framing effects in social media and (2) providing ideas for drafting health-promoting messages with a high degree of influence.

First, the use of gain-framed messages in the health themes of childhood obesity (*P*<.001), smoking (*P*=.03), and cancer (*P*<.001) can significantly improve the influence of health-promoting messages (Table 2). Rothman and Salovey [46] and Rothman et al [47] divided health behaviors into prevention and detection behaviors according to the risk perception of individuals. Preventive behaviors include exercise, quitting smoking, eating healthy, and using sunscreen. They believed that gain-framed messages were more persuasive in promoting disease prevention behaviors. Goal-framing effects based on prospect theory have also revealed that factually equivalent messages have different levels of persuasiveness depending on the frame adopted by the messages [48]. Gallagher and Updegraff [49] believed that gain-framed health-promoting messages stimulated more information processing and better subsequent memory. Furthermore, many previous investigations support our conclusion. A cross-sectional study of 592 caregivers of preschool children found that gain-framed messages could significantly improve the acceptance of information by caregivers [25]. Romanowich and Lamb [50] posited that health education using gain-framed messages could be more useful for nonsmokers. A qualitative survey of African American adolescents by Satia et al [51] indicated greater consistency with gain-framed cancer prevention messages. Most research on the framing effects of health-promoting messages have been conducted by questionnaires or interviews [15]. However, the real world involves people observing information and making decisions in a complex environment [52]. This study further confirmed that gain-framed messages are a favorable strategy in the dissemination of health-promoting messages in everyday life.

Second, the use of statistical expression in the health themes of childhood obesity (*P*=.02), smoking (*P*=.002), and cancer (*P*<.001) can significantly improve the influence of health-promoting messages (Table 2). Statistical expressions refer to health-promoting messages with numerical content [53]. Nonstatistical expressions denote health-promoting messages without any precise numbers and are usually used in the description of examples and stories [53]. No conclusion has been reached about the persuasiveness of these two expression types [34]. A meta-analysis [23] and an investigation of 1270 participants [54] found that statistical messages were more convincing than narrative ones. A message that combines narrative and statistical expression is more convincing than one using either narrative or statistical expression alone. We hypothesized that adding statistical expressions to health-promoting messages when describing health behaviors and consequences could create more active engagement toward those messages and earn them more retweets, comments, and likes. Another meta-analysis also revealed that statistical expression has a stronger impact on beliefs and attitudes than narrative expression and that statistical expressions, beliefs, and attitudes are mainly related to cognitive responses [55]. Wong et al [39] combined numerical framing effects and prospect theory and verified that precise numbers could more easily represent the probability of risk. We believe this finding may explain why people pay more attention to health-promoting messages that contain statistical expressions.

Third, the use of videos significantly improved the influence of health-promoting messages only for messages regarding smoking (*P*=.009). A review suggested that compared with text alone, adding pictures that are closely related to the written text can significantly improve the attention to and recall of health education information [56]. However, Houts et al [56] noted that great care should be taken when including picture materials in health messages so that the audiences can understand the key points of the message without being distracted by irrelevant details. Levie and Lentz [57] posited that pictures not closely related to the text have no beneficial effect on comprehension. Furthermore, the impact of using videos on health-promoting messages may be uncertain. Occa and Suggs [58] found that videos had a positive impact when communicating breast cancer information to 194 Italian women. Conversely, Xie [59] suggested that there was no significant difference in risk perception caused by words and sounds. We suggest that publishers add appropriate pictures and videos as much as possible when making health-promoting messages.

Fourth, we believe that accounts from organizations in health fields should release health-promoting messages more actively. People tend to trust universities and official institutions more than other types of organizations [60]. Furthermore, people consider private doctors, medical universities, and governments the most trusted sources of health messages [44,45]. As shown in Table 1, the proportion of accounts from individual users was higher than for organizational users, and users engaged in the health field outnumbered those in nonhealth fields. Specific audiences are more willing to believe that the most reliable information is provided by accounts from a health field [24]. Thus, ordinary users and organizations from health fields must participate in the dissemination of health-promoting messages.

Fifth, we found that health-promoting messages account for a very small proportion of the social media posts related to the 3 health themes. We thought this may be related to four main reasons. First, the definition of a health-promoting message was a message that included both health behaviors and health outcomes [15], so we excluded some messages, such as messages only referring to the cause of a disease. Second, in a health topic, the amount of social content was often much larger than the amount of health professional content [61]. Third, we found that the celebrity effect exists in Weibo health themes. If a celebrity died of cancer, there would soon be a lot of cancer-related messages on Weibo. However, there was a lack of clear health guidance in these messages. We excluded a lot of these kinds of eye-catching messages. Fourth, even under the theme of health, a lot of messages on social media were still related to advertising [62]. We excluded the messages that contained advertising. This result reflects reality. There were few health-promoting messages published on Weibo about childhood obesity, smoking, and cancer. In the health field, many researchers have confirmed that health-promoting messages using framing effects can stimulate people's health awareness [63] and improve their willingness to prevent and treat health conditions [20,49]. Health promotion messages including health behaviors and health outcomes should be widely used. Our research suggests that professionals in health fields should be more active in publishing health-promoting messages on social media.

### Limitations

This study has several limitations. First, a small sample size was analyzed in this work. We needed to explore the impact of goal-framing effects on the influence of health-promoting messages. Accordingly, we included only health-promoting messages, such as those about behaviors and consequences [15], thereby limiting the writing template for such messages. This approach did not fully incorporate all health messages and may have produced errors. Moreover, this research only focused on certain health-promoting messages to fill the gaps in the literature. Second, we formulated the definition of a Weibo message’s degree of influence according to the literature [41,42] and from expert advice, and we used only 3 indexes: retweets, comments, and likes. The number of users who viewed a message is an excellent index to evaluate the degree of influence, but restrictions of the Weibo platform meant that we could not ascertain the number of views for every message. If the Weibo platform cancels this restriction in the future, then views should be included in the evaluation index. Third, we did not evaluate whether the pictures and videos added in the Weibo health-promoting messages accurately matched the points of the message. Such an omission may have affected our results. We still suggest that the publisher add appropriate pictures and videos as much as possible to health-promoting messages. In the future, researchers can further examine the impact of pictures and videos on health-promoting messages on social media.

### Conclusions

In this study, we identified the factors that could affect the degree of influence of health-promoting messages on the Sina Weibo platform. A total of 389 health-promoting messages were included in this work. The use of gain-framed messages and statistical expressions could improve the influence of messages for all 3 themes (ie, childhood obesity, smoking, and cancer). Although adding pictures and videos to messages did not significantly improve the influence of messages about childhood obesity and cancer, we still contend that adding appropriate pictures and videos as much as possible when producing health-promoting messages is a good strategy. We encourage users from organizations in health fields to release more health-promoting messages. When public health institutions and professionals release such messages, the framework, organization, and content of the messages must be considered. In this way, health-promoting messages may become more influential.

## Appendix

Multimedia Appendix 1 Coding principle for the health-promoting messages.
