# Supplementary material for: Factors Associated With Influential Health-Promoting Messages on Social Media: Content Analysis of Sina Weibo
Source: JMIR Med Inform. 2020 Oct 9;8(10):e20558. doi: 10.2196/20558 (PMC7584979; doi:10.2196/20558)
Supplement: Multimedia Appendix 1 [file medinform_v8i10e20558_app1.docx]

## Appendix A

## Coding Principle for the Health-promoting Messages

## Originality: The mechanism of Weibo allowed users to add new content when they retweet other’s messages. Others can read both original and new content. Therefore, ascertaining whether the original content or the new content had the influence effect is impossible. Consequently, we included only the original Weibo messages. If a Weibo message was retweeted in other's messages, then a retweet flag would appear, thereby making such messages readily identifiable to the reviewers.

## Calculation of influence degree: We use RTs, Comments, and Likes to evaluate the influence score of each Weibo message. Given the variation in the time costs and credit costs of Comments, Retweets, and Likes, weights must be assigned to different projects.

## The definition formula of a Weibo message influence degree is as follows:

## Is = α𝐿w+𝛽Rw+𝛾𝐶w.

## Is is the influence score of a Weibo message, Lw is the number of Likes, Rw is the number of RTs and Cw is the number of Comments. α, 𝛽, and 𝛾 are the weights assigned to these three projects. According to the research of Xiong and Wu, and the discussion of an expert panel, we set α, 𝛽, and 𝛾 as 0.2, 0.365, and 0.435, respectively.

## Conversely, the definition formula of the final influence degree is

## Is = 0.2𝐿w + 0.0.365Rw + 0.435𝐶w.

## We calculated the total influence score of all messages in an account within 30 days and then divided it by the number of messages required to obtain the average influence score of the account. A health-promoting message published by the account and bearing influence score greater than or equal to the average influence score of the account is coded as “high influence message”. By contrast, a message with influence score that is less than the average influence score of the account is coded as “low influence message”.

## Frame properties (Fp): Following literature analysis, Jonathan et al. posited that a health-promoting message can be framed according to the beneficial consequences of healthy behavior (gain-framed message) or the harmful consequences of unhealthy behavior (loss-framed message). To judge whether a behavior is healthy or unhealthy, we can use the clinical guidelines of related topics to regulate such assessment. We collected clinical guidelines on Childhood obesity, Smoking, and Cancer to determine whether a behavior is healthy. After literature analysis and expert group discussion, we classified the frame properties into three categories:

## a) Recommended behaviors in clinical guidelines + Positive results on the body = Gain-framed message,

## b) Behaviors not recommended or prohibited in clinical guidelines + Negative results on the body = Loss-framed message,

## c) Situations including both a) and b).

## We coded gain-framed messages (a) as 0, loss-framed counterparts (b) as 1 and neutral-framed ones (c) as 2 (See Table A.1).

## Message source (Ms): The title authentication column of Sina Weibo encompasses user authentication, including the user authentication domain and user classification (as an organization or institution). The verification system can ensure data authenticity. We coded the messages released by ordinary users (Health field) as 0, ordinary users (Non health field) as 1, organizations (Health field) as 2 and organizations (Non health field) as 3 (See Table A.1).

## Expression type (Et): Statistical evidence refers to the proof provided by quantitative or digital information. We read health-promoting messages to check if they contain accurate numerical descriptions. For instance, ‘350ml of juice can contain up to 9 teaspoons of sugar’ is a precise expression. We coded the messages of statistical expression as 0 and of non- statistical expression as 1 (See Table A.1).

## Picture and video assistance (Pa and Va): We obtained the results by observing the output of the crawler program. We coded the messages with picture assistance as 0 and without picture assistance as 1. We also coded the messages with video assistance as 0 and without video assistance as 1 (See Table A.1).

## As shown in Table A.1, we randomly selected three health-promoting messages of different themes to show the coding principles.

## Table A.1 Display of coding results and messages content.

| Health Theme | RTs | Comments | Likes | Influence score | Fp | Ms | Et | Pa | Va |
| --- | --- | --- | --- | --- | --- | --- | --- | --- | --- |
| Childhood Obesity | A systematic review and meta-analysis led by St. Michael's United Health Hospital in Toronto found that children who consumed whole milk were 40% less likely to be overweight or obese than those who consumed low-fat milk. | | | | | | | | |
| Coding | 15 | 29 | 61 | 30.29 | 0 | 0 | 0 | 1 | 0 |
| Smoking | Recently, a study from the University of Nottingham showed that smokers have a higher risk of influenza than non-smokers Five More than times. | | | | | | | | |
| Coding | 5 | 4 | 9 | 5.365 | 1 | 0 | 0 | 1 | 0 |
| Cancer | Get moving: new research finds that exercise can reduce the risk of cancer. | | | | | | | | |
| Coding | 6 | 0 | 5 | 3.19 | 0 | 3 | 1 | 0 | 1 |
